# Evolution of Volatile and Phenolic Compounds during Bottle Storage of Merlot Wines Vinified Using Pulsed Electric Fields-Treated Grapes

**DOI:** 10.3390/foods9040443

**Published:** 2020-04-06

**Authors:** Mylene Ross Arcena, Sze Ying Leong, Martin Hochberg, Martin Sack, Georg Mueller, Juergen Sigler, Patrick Silcock, Biniam Kebede, Indrawati Oey

**Affiliations:** 1 Department of Food Science, University of Otago, Dunedin 9054, New Zealand; mylene.arcena@postgrad.otago.ac.nz (M.R.A.); sze.leong@otago.ac.nz (S.Y.L.); pat.silcock@otago.ac.nz (P.S.); biniam.kebede@otago.ac.nz (B.K.); 2 Riddet Institute, Palmerston North 4442, New Zealand; 3 Institute for Pulsed Power and Microwave Technology, Karlsruhe Institute of Technology, Eggenstein-Leopoldshafen, 76344 Karlsruhe, Germany; martin.hochberg@kit.edu (M.H.); martin.sack@kit.edu (M.S.); georg.mueller@kit.edu (G.M.); 4 State Institute of Viticulture and Oenology, 79100 Freiburg, Germany; juergen.sigler@wbi.bwl.de

**Keywords:** pulsed electric fields, wine, storage, volatile fingerprinting, phenolics profiling, merlot, multivariate data analysis

## Abstract

This study aimed to elucidate changes in volatile, phenolic, and oenological profiles of wines vinified from Pulsed Electric Fields (PEF)-treated and untreated Merlot grapes during bottle storage of up to 150, 90, and 56 days at 4 °C, 25 °C, and 45 °C, respectively, through chemometrics technique. Wines produced from untreated grapes and those PEF-treated at four different processing conditions (electric field strength 33.1 and 41.5 kV/cm and energy inputs between 16.5 and 49.4 kJ/kg) were used for the bottle storage study. Results showed that hydroxycinnamic and hydroxybenzoic acids in all stored wines, regardless vinified from untreated and PEF-treated grapes, increased as a function of time and temperature, while anthocyanins and selected esters (e.g., ethyl butanoate) decreased. Extreme storage temperature, at 45 °C particularly, resulted in a higher amount of linalool-3, 7-oxide in all stored wines. After prolonged storage, all wines produced from grapes PEF-treated with four different processing conditions were shown to favor high retention of phenolics after storage but induced faster reduction of anthocyanins when compared to wines produced from untreated grapes. Moreover, some volatiles in wines vinified using PEF-treated grapes, such as citronellol and 2-phenylethyl acetate, were found to be less susceptible towards degradation during prolonged storage. Production of furans was generally lower in most stored wines, particularly those produced from PEF-treated grapes at higher energy inputs (>47 kJ/kg). Overall, PEF pre-treatment on grapes may improve storage and temperature stability of the obtained wines.

## 1. Introduction

Pulsed electric fields (PEF) as a pre-maceration treatment has been shown to significantly improve the extraction of anthocyanin and phenolic compounds from red grape skins during vinification [1]. As a result, the finished wines produced from PEF-treated grapes have been consistently reported to contain a higher number of phenolic compounds compared to those wines vinified using non-PEF-treated grapes [2]. It is important to note that phenolic content and chemical composition can significantly contribute to the organoleptic properties of wines, such as color perception, taste, mouthfeel, and aroma [3]. These wine qualities experience further changes over time as volatile and phenolic composition evolves during bottle storage prior to consumption. The evolution of wine quality during this latter period can be described as maturation, peak quality, and deterioration [4]. During maturation, wine color, flavor, and stability are expected to refine and reach the peak quality, which is then followed by quality deterioration. Storage conditions, such as time and temperature, together with the initial composition of the wine prior to storage, are important factors that can influence the rate of quality changes [3,5,6]. Hence, investigating these factors can determine the stability of wines made from PEF-treated grapes (thereafter referred as “PEF wines”) and might be helpful in establishing appropriate storage conditions to preserve their quality.

A few studies have evaluated the quality of PEF wines, solely on Cabernet Sauvignon for the changes in the overall phenolic content and color characteristics, during aging in bottles [7,8] and oak barrels [9]. Their results showed that total polyphenol index (measured using Folin–Ciocalteu colorimeter assay), total monomeric anthocyanins, as well as specific group of phenolics from flavan-3-ols, hydroxycinnamic acids, and flavanols in PEF wines were significantly reduced after bottle ageing for 12 months at 18 °C, but the reduction of each of these occurred at different rates compared to the wines produced from untreated grapes (thereafter referred as “control wines” or “non-PEF wines”) [7]. Overall, aged PEF wines contained a higher number of anthocyanins and phenolics at the end of the ageing period, probably because PEF wines had a higher initial level of these compounds at the beginning of the wine ageing experiment [7]. Findings from previous studies have demonstrated that the rate of phenolic reduction during bottle ageing or storage between PEF and control wines varied greatly between different phenolic compounds. This clearly revealed that a PEF pre-treatment applied to grapes at the onset of vinification may play a key role in modulating the evolution and stability of various chemical constituents important for wines’ quality when they are stored in bottle. To date, investigation of the storage stability of PEF wines is limited to one grape cultivar (Cabernet Sauvignon) and are based solely on the changes of pre-determined phenolic compounds and attributes [7,8,9]. These studies have considerably overlooked the synergistic and cascading effects of a number of phenolic compounds, color, and volatility of aroma compounds as a function storage temperature and duration.

The purpose of this research was to study the overall evolution that occurs during the storage of Merlot wines vinified from PEF-treated grapes by employing multiplatform analytical approach through combining targeted and untargeted approaches [10]. Targeted analytical methods were employed to investigate prior selected attributes such as phenolic compounds and color and oenological properties. An untargeted gas chromatography-mass spectrometry (GC-MS)-based fingerprinting method was implemented with the aim of detecting as many volatile compounds present as possible in the wine headspace fraction. The huge data obtained with the targeted and untargeted methods was fused and analyzed with multivariate data analyses, such as principal component analysis (PCA) and partial least square-regression (PLS-R). The grapes were treated with PEF at four different processing conditions (electric field strength 33.1 and 41.5 kV/cm and energy inputs between 18.9 and 49.4 kJ/kg) known to be effective in extracting a wide range of anthocyanins from Merlot grapes based on our previous study [1]. Therefore, gaining an understanding regarding the storage stability of Merlot wines vinified with grapes PEF-treated with the aforementioned processing condition becomes necessary. Important changes over storage time at different storage temperatures for each wine (either PEF or non-PEF wines) were determined through the selection of discriminant markers.

## 2. Materials and Methods 

### 2.1. Grapes

A batch of Merlot grapes (*Vitis vinifera* var. Merlot) amounting to five tonnes was harvested from the Hawke’s Bay region (North Island, New Zealand). The grapes were harvested at the end of March 2016 at the optimum ripening stage (18 °Brix) and transported overnight to winery for PEF treatment and vinification.

### 2.2. PEF-Assisted Vinification of Merlot Grapes

#### 2.2.1. PEF Treatments

Prior to PEF treatment, grapes were destemmed and crushed into musts. All PEF treatments on grapes were achieved using the KEA-WEIN electroporation device (Karlsruhe Institute of Technology, Karlsruhe, Germany) with a throughput of 500 kg/h and the capability to generate high intensity electric field strengths (up to 42 kV/cm) at a wider range of total energy inputs (up to 50 kJ/kg). The device comprises a 6-stage Marx generator (up to 33 kV charging voltage per stage) connected to the electroporation reactor, which is made up of two parallel stainless-steel electrodes of 3.5 cm apart, allowing a quasi-homogenous field distribution [11]. The length of the reactor measured 4.7 cm, resulting in a cross-section area of 12.5 cm^2^ and a volume of 57.6 cm^3^. To avoid flashover occurrence inside the reactor, musts were pressurized to approximately 0.2 MPa (2 bar) above ambient pressure. In this study, grapes were subjected to four different PEF processing conditions (PEF2, 5, 6, and 8) with varying intensities of electric field strength and total specific energy inputs based on the findings from previous study [1] to achieve improvements in anthocyanin extraction immediately after PEF application. Grapes that received PEF2 and PEF5 treatments were subjected to fixed electric field strength of 33.1 kV/cm at the frequency of 10 Hz and 25 Hz, respectively. For PEF6 and PEF8 treatments, fixed electric field strength of 41.5 kV/cm at the frequency of 5 Hz and 15 Hz were applied on Merlot respectively. The specific energy input was 18.90, 47.25, 16.47, and 49.40 kJ/kg for samples treated at PEF2, 5, 6, and 8, respectively. Moreover, a batch of untreated grapes was pumped through KEA-WEIN without PEF application (thereafter referred as “PEF0”). 

Pulse current in the reactor was measured through a Rogowski coil and was displayed on a connected oscilloscope. The measured pulse current, together with the circuit parameters and the area of reactor, was used to determine the electric field strength. During the course of each PEF treatment, the temperature of grape must was monitored at both the inlet (T_in_) and outlet (T_out_) of the reactor. The total specific energy input applied to grapes for each PEF treatment takes into consideration the temperature difference (T_out_-T_in_) and the specific heat capacity of grapes (i.e., 3.5 kJ/K/kg) determined calorimetrically by heating up 1 kg of grapes by 5 K [11]. After PEF processing, all grape musts were fermented into wines under commercial vinification practice involving maceration–alcoholic and malolactic fermentations. 

#### 2.2.2. Vinification Process

Merlot wines were elaborated following the standard red wine making procedure in a commercial winery. All grape musts were vinified in 500 L open top stainless-steel fermenters. Potassium metabisulphite (50 ppm) was added within 24 h prior to the inoculation of *Saccharomyces cerevisiae* wine yeasts. Alcoholic fermentation occurred between 16.5 °C and 25.5 °C, and was completed within seven days. Afterwards, the fermenting must was pressed and the resulting wine was contained in 50 L metal kegs, where malolactic fermentation was initiated upon immediate inoculation of malolactic bacteria. Approximately six weeks after malolactic fermentation at 12 ± 1 °C, the wines were racked off the lees and combined with 30 ppm potassium metabisulphite. Then, the finished wines were bottled and tightly sealed in individual screw capped bottles (325 mL per bottle). Merlot wines made from differently PEF-treated grapes are named according to the PEF treatment they received before fermentation, i.e., PEF2, 5, 6, and 8 wine refers to wine vinified using grapes pre-treated with PEF2, 5, 6, and 8, respectively, while “control wine” or “non-PEF wines” refers to wines vinified with untreated grapes (or PEF0).

### 2.3. Storage Experiment of Bottled PEF Wines under Different Temperatures as a Function of Time 

For each type of wine (PEF0, 2, 5, 6, and 8), a total of 21 bottles of wine were randomly allocated to be stored upright under dark conditions at three storage temperatures (4 °C, 25 °C, and 45 °C; seven bottles per temperature). A kinetic approach [6] to monitor the time evolution of volatile and phenolic compounds in Merlot red wines vinified with untreated and PEF-treated grapes as a function of temperature was employed in this study. Each bottle was opened for sampling at predefined storage time intervals for each storage temperature. The storage experiment at three different temperatures started on the same day (Day 0). Three bottles of wine from each wine type were sampled on the day (labelled according to the PEF treatment and sampling day “P0D000, P2D000, P5D000, P6D000, and P8D000” samples), while the remaining 18 bottles were transferred into their allocated storage temperatures. Wines that were stored in a 4 °C temperature-regulated walk-in cold room and were sampled at day 0, 60, 90, and 150. For wines stored at 25 °C temperature control room, wine was sampled at day 0, 14, 30, 60, 90, and 120. Wines stored in a 45 °C incubator (Jeiotech IB-15G, Lab Companion, Seoul, South Korea) were sampled more frequently (i.e., every seven days) at day 0, 6, 14, 28, and 56, as chemical changes in red wine tend to occur at a faster rate at elevated temperatures [6]. On the day of sampling, wine bottles were removed from the incubators (especially for 25 °C and 45 °C) and placed in a 4 °C temperature-controlled room for no more than 2 h before opening the screw capped bottles. Then, four 35 mL aliquots from newly opened bottles were immediately frozen using liquid nitrogen and stored at −80 °C until further analysis of volatile and phenolic profiles. Aliquots were labelled according to the PEF conditions followed by the number of storage days (e.g., D000 and D060 for 0 day/initial sampling and 60 days of storage, respectively). 

### 2.4. Determination of the Oenological Properties for Stored PEF Wines 

On the day of sampling, each wine sample was analyzed for titratable acidity (TA), pH, and color properties such as color intensity (CI) and the color space values (lightness (L*), green to red (a*), blue to yellow (b*), chroma (C*, saturation), and hue (h°, hue) The measurement of pH was performed using a pH meter (Hannah Instruments pH 209, Inc., Woonsocket, RI, USA). TA (expressed as gram of tartaric acid per liter of wine) was determined by titrating 5 mL of wine sample against 0.1 N sodium hydroxide (NaOH; VWR Prolabo, Leicestershire, England) to an endpoint of pH 8.2 ± 0.05. CI was determined by adding up the absorbance of samples at wavelengths 420, 520, and 620 nm [12], measured using a UV/vis spectrophotometer (Ultrospec 3300 Pro, Amersham Biosciences, Sweden). Prior to spectrophotometric measurement, wine samples were centrifuged (9335 × *g*, 5 min; IEC Micromax Centrifuge fitted with IEC 851OF Fixed-Angle Rotor, International Equipment Company, Needham Heights, MA, USA), and the supernatant was diluted adequately. Color space values represented as the CIELab coordinates, L* (lightness), a* (green to red), and b* (blue to yellow), were measured using HunterLab colorimeter (MiniScan EZ 4500S, Reston, VA, USA). Chroma (C*, saturation) and hue angle (h°) were further derived from the measured CIELab coordinates. TA and pH were measured in triplicates while color measurements were done in four replicates.

### 2.5. Determination of the Volatile Fingerprints for Stored PEF Wines

Untargeted analysis of the volatile profile of the wine samples were performed according to the volatile extraction and gas chromatography (GC) methods conducted by Arcena et al. [13]. Volatile fingerprinting was performed using the Agilent 6890N gas chromatography system connected to an Agilent Mass Selective Detector 5975 VL. Wine samples (prepared as 25% (v/v) in deionized water) mixed with 2.5 g sodium chloride (Merck, Darmstadt, Germany) were prepared in 20 mL headspace glass vials tightly capped and sealed with silicon septa screw caps coated with polytetrafluoroethylene (PTFE). Sample vials were equilibrated with agitation at 40 °C for 5 min. Using pre-conditioned divinylbenzene/carboxen/polydimethylsiloxane (DVB/CAR/PDMS) coated solid-phase microextraction (SPME)fiber (50/30 μm, Supelco Co., Bellefonte, PA, USA), headspace volatiles from the samples were extracted at 40 °C for 30 min and desorbed at 230 °C in a splitless mode for 5 min. Four independent extractions were performed for each sample. Volatile compounds were separated through a ZB-Wax column (60 m × 0.32 mm inner diameter × 0.5 μm film thickness; Phenomenex) at a constant flow rate of 1 mL/min. The column was heated according to the following temperature ramp: Holding 50 °C for 5 min; raising at 5 °C/min to reach 210 °C; raising at 10 °C/min to reach 240 °C and holding for 5 min; then, cooled down to 50 °C. Mass spectrometry (MS) was operated with electron ionization set at 70 eV, a scanned mass range of 30–300 m/z, MS ion source held at 230 °C, and MS quadrupole mass analyzer at 150 °C. To monitor any systematic error and fiber carry over, quality control and blank samples were injected every ten GC-MS analysis.

Based on previous works [14,15], GC-MS total ion chromatograms were pre-processed via an automated mass spectral deconvolution and identification system (AMDIS; Version 2.72, 2014, National Institute of Standards and Technology (NIST), Gaithersburg, MD, USA) and mass profiler professional (MPP, version 14.9.1, 2017, Agilent Technologies, Mulgrave, VIC, Australia). Peaks were identified and validated through the following three criteria: (a) match and reverse match >90% of the spectral of separated peaks with NIST spectral library (NIST14, version 2.20, National Institute of Standards and technology, Gaithersburg, MD, USA); (b) comparison of the experimental retention index (RI) with literature RI; and (c) matching retention time with authentic standards for each chemical group analyzed under the same GC oven program.

### 2.6. Determination of Anthocyanins and Phenolic Composition for Stored PEF Wines 

Targeted measurement of phenolic compounds in stored wines were conducted with a minimal extraction procedure. After overnight thawing in 4 °C dark room, wine samples were centrifuged (9335 g, IEC Micromax Centrifuge) for 5 min, filtered (0.45 µm cellulose syringe filter), and 5 µL of sample was directly injected into the reversed-phase Kinetex C_18_ column (100 × 4.6 mm i.d., 2.6 µm pore size, 20 °C; Phenomenex, Torrance, CA, USA) on an Agilent 1200 system (Agilent Technologies, Palo Alto, CA, USA) connected to a diode-array detector. Solvents include 10% (v/v) formic acid (Merck, Darmstadt, Germany) in acetonitrile (solvent A; VWR International, Monroeville, PA, USA); and 10% (v/v) formic acid in deionized water (solvent B). Gradient elution using both solvents ran at a flowrate of 1 mL/min in the following conditions: 0–12.5 min, 96.7% solvent B; 12.5–13.5 min, 64.3% solvent B; 13.5–14.5 min, 96.7% solvent B; and 14.5–20 min, 96.7% solvent B. Phenolic compounds (i.e., anthocyanins, flavonols, flavanols, stilbenes, hydroxybenzoic, and hydroxycinnamic acids) were identified and quantified at 520, 360, 325, and 280 nm based on the retention times of the known standards (≥95% purity) and the external calibration curve of the respective authentic standard diluted in methanol. Results were then expressed as mg phenolic per liter of wine. Four sample replicates were analyzed.

### 2.7. Multivariate Data Analysis through Partial Least Squares-Regression (PLS-R)

Data on the oenological properties, color, volatile, and phenolic profiles were pooled together into one data table for multivariate data analysis (MVDA). To investigate the changes as a function of storage time, data sets from samples of the same PEF treatment stored at the same temperature were grouped together to produce 15 data set groups (i.e., 5 PEF treatments × 3 storage temperatures). MVDA of these merged data sets were carried out as specified by Kebede et al. [14] and Buvé et al. [15]. Using SOLO software (Version 8.6, 2018, Eigenvector Research, Wenatchee, WA, USA), principal component analysis (PCA) and partial least squares-regression (PLS-R) were performed. Based on PCA bi-plots, outliers and groupings were detected. Afterwards, PLS-R was conducted to describe the effect of storage time. For PLS-R, the metabolites and storage time were designated as X- and continuous Y-variables, respectively. Cross validation was applied to select the optimum number of latent variables (LV) that explain maximum variance/information in the data while maintaining error at a minimum in a model. From this model, PLS-R bi-plots were constructed using Origin Pro 2018 software (Origin Lab Corporation, Northampton, MA, USA). This is a graphical representation of the differences between samples of varying storage time. To identify metabolites significantly affected by storage time, variable identification coefficients (VID) were calculated as the correlation coefficient of the original X-variable (metabolites) to the Y-variable (storage time) based on the PLS-model. Metabolites with the minimum VID coefficient of |0.800| were designated as discriminant markers (significantly changing during storage). Afterwards a statistically significant difference for these discriminant markers between different PEF wines was calculated using Student’s *t*-test for single comparison, as well as using an analysis of variance (ANOVA) for multiple comparisons followed by post-hoc Tukey’s honestly significant difference (HSD) test, performed using IBM SPSS Statistics version 25 (IBM Corporation, New York, USA). The criterion employed for a statistical significance of the difference was *p* < 0.05.

## 3. Results and Discussion

In this study, the effect of storage time and temperature on wines from differently PEF-treated and untreated Merlot grapes were extensively examined. The untargeted headspace-GC-MS fingerprinting procedure enabled the detection of up to 75 volatile compounds from a wide range of chemical families (e.g., ester, aldehyde, alcohol, terpene, etc.). Furthermore, multiple targeted analytical procedures were applied to measure 29 phenolic compounds together with their color and oenological properties. All data obtained with the targeted and untargeted approaches were combined into a single data matrix and modelled using multivariate data analysis, and finally identifying the discriminant compounds and properties considerably affected by both the storage time and temperature.

### 3.1. Visualization of the Evolution of the Oenological, Volatile, and Phenolic Fingerprints for the Bottled PEF Wines at Three Temperatures as a Function of Storage Time

**Figure 1:**
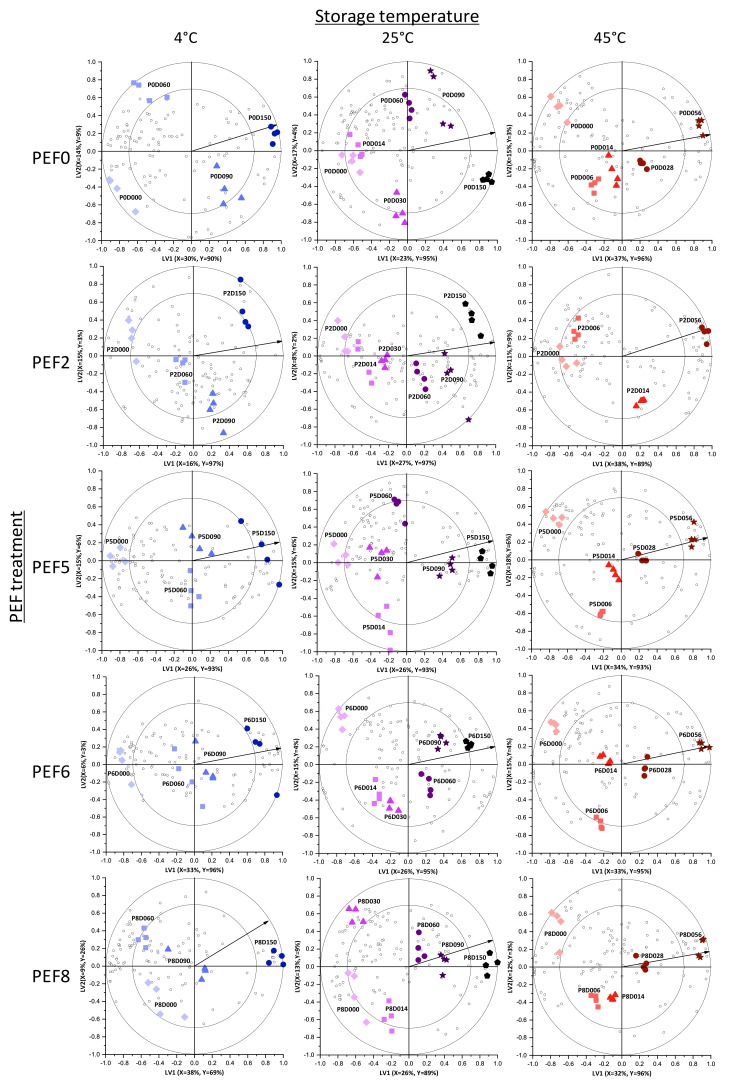
Partial least squares-regression (PLS-R) bi-plots illustrating the changes as a function of storage time on the volatile fingerprint, phenolic profile, and basic oenological properties of Merlot wines from pulsed electric fields (PEF)-treated (PEF2, 5, 6, 8) and untreated (PEF0) grapes. Classes based on storage time are represented as colored solid shapes and labelled (e.g., P0D000) according to PEF treatment (e.g., P0 for PEF0) and storage days (e.g., D000 for day 0). Metabolites are drawn as open circles. Vectors signify the correlation loadings for the categorical Y-variable. The percentages of X- and Y-variances explained by each latent variable (LV) are specified on the respective axes.

The changes in volatile, phenolic, and oenological properties of wines vinified from PEF during storage were studied using partial least squares-regression (PLS-R). For PLS-R modelling, the detected compounds were considered as X-variables, and storage time was considered the continuous Y-variable. Based on the PLS-R model, bi-plots were constructed to visualize the changes as a function of storage time for each condition. Figure 1 shows 15 bi-plots (for five PEF processes and three storage temperatures). In all 15 bi-plots, storage time clearly separated and positioned the samples from the initial (left side of bi-plot) to the last sampling point (right side of bi-plot). This is further indicated by the vectors that represent the correlation loading for the Y-variable (storage time). The long vectors indicate that the two chosen LVs explained a higher percentage of Y-variation [16]. This variation is driven by the significant changes in the concentrations of the metabolites, which are represented as the open circles on the bi-plots. The relationship between the metabolites and storage time can be interpreted based on their projection relative to the vector and the ellipses, where inner and outer ellipses signify correlation coefficients of 70% and 100%, respectively. Those metabolites positioned beyond the inner ellipse and on the direction of the vector increased as a function of storage time. On the other hand, those metabolites found beyond the inner ellipse but directed opposite the vector decreased during storage. Meanwhile, metabolites inside the inner ellipse can be considered somewhat stable during storage. In all stored wines, most metabolites reduced over time.

VID coefficients of the attributes were calculated to identify those metabolites affected by storage time at each storage temperature in each PEF condition, as reflected by those metabolites (open circles, Figure 1) closely positioned to every wine sample (colored solid shapes, Figure 1) on the PLS-R bi-plot (Figure 1). Table 1, Table 2, Table 3, Table 4 and Table 5 list metabolites significantly increasing and decreasing as a function of storage time on stored wines from PEF-treated and untreated Merlot grapes.

### 3.2. Key Changes in the Color, Oenological, Volatile, and Phenolic Fingerprints for the Bottled PEF Wines at Three Different Storage Temperatures as a Function of Time

The effect of storage time and temperature on Merlot wines vinified from untreated and PEF-treated grapes can be identified through similar key trends among the markers (based on Figure 1 and Table 1, Table 2, Table 3, Table 4 and Table 5) across the wine samples, which are discussed in Section 3.2.1, Section 3.2.2, Section 3.2.3 and Section 3.2.4. By looking at the VID coefficients, one can interpret the amount of detected compounds. Compounds with positive VID coefficients are detected at high amounts in that specific sample compared to the other samples and vice versa. However, to have a better understanding on the overall changes during storage, discriminant markers distinct to certain wines stemming from the differences in the initial composition a result of PEF-pre-treatment are also discussed in Section 3.2.5.

#### 3.2.1. Increases in the Hydroxycinnamic and Hydroxybenzoic Acids Over Storage Time

**Figure 2:**
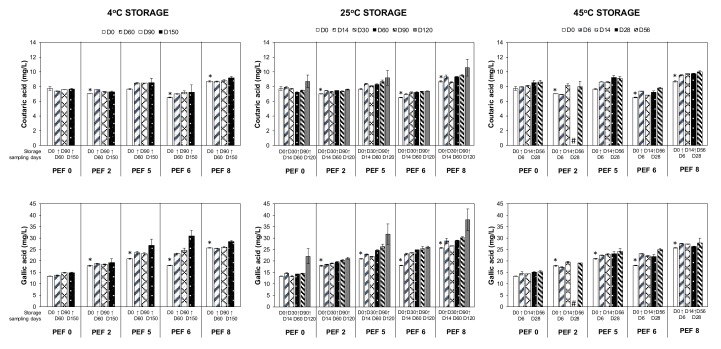
Increases in the concentration of selected hydroxycinnamic and hydroxybenzoic acids in Merlot PEF wine samples over storage time at 4 °C, 25 °C, and 45 °C. Data presented as mean ± standard deviation (*n* = 4). Significant difference (*p* < 0.05) comparing the concentration of the analytes between PEF wines (PEF2, 5, 6, and 8) from non-PEF wines (PEF0) at the start of the storage trial (day 0, D0 white bar) are indicated with asterisk (*). # indicates data not collected.

For all storage temperatures, phenolic acids (i.e., hydroxycinnamic and hydroxybenzoic acids) increased over time for all PEF wines (Figure 2). The higher concentration of hydroxycinnamic acids can be attributed to the hydrolysis of tartaric esters present in the young wines into their corresponding free acids, such as caffeic acid and coumaric acids from caftaric acid and coutaric acid, respectively [4,17,18]. This trend has been observed in red wines from both PEF-treated [7] and untreated grapes [18,19]. The surge in hydroxybenzoic acids, particularly gallic acid, protocatechuic acid, and syringic acid, may have resulted from the cleavage of anthocyanins during wine storage (e.g., malvidin 3-*O*-glucoside may lead to production of syringic acid) [17]. Also, the hydrolysis of galloylated tannins in the wines may have contributed to gallic acid concentrations [20]. A study done by Ferrer-Gallego et al. [21] has shown that the presence of both hydroxycinnamic and hydroxybenzoic acids, in a mixture, can contribute towards the astringent perception. Therefore, the sensory implication in Merlot wine due to increases in the hydroxycinnamic and hydroxybenzoic acids over storage time could be significant. 

#### 3.2.2. Decreases in the Anthocyanins and Color Properties over Storage Time

**Figure 3:**
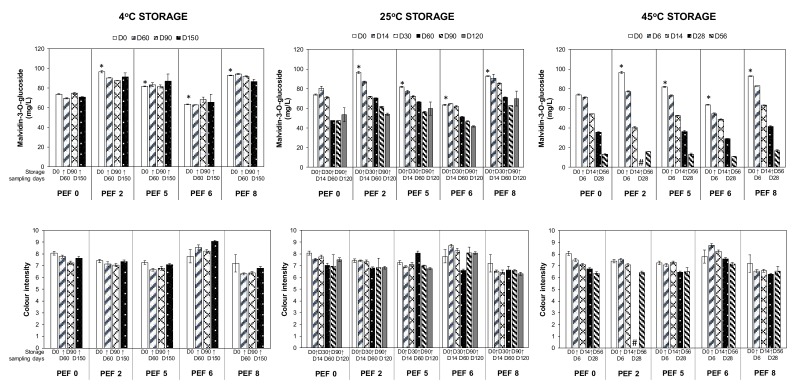
Decreases in the concentration of selected anthocyanin and color properties in Merlot PEF wine samples over storage time at 4 °C, 25 °C, and 45 °C. Data presented as mean ± standard deviation (*n* = 4). Significant difference (*p* < 0.05) comparing the concentration of the analytes between PEF wines (PEF2, 5, 6, and 8) from non-PEF wines (PEF0) at the start of the storage trial (day 0, D0 white bar) are indicated with asterisk (*). # indicates data not collected.

A reduction in the anthocyanins accompanied by the dulling of color over storage time, particularly at higher storage temperatures, was expected (Figure 3). Apart from experiencing degradation, oxidation, and cleavage into hydroxybenzoic acids, monomeric anthocyanins can participate in reactions contributing to the color stability of the wine including self-association, co-pigmentation, polymerization, and formation of new pigments, which has been reported by several authors on storage of red wines [3,22,23,24]. These changes involving anthocyanins explain the shift from a red–purple color imparted by the monomeric anthocyanins in the young wine to the brick-red hues resulting from a combination of new pigments and complexes with anthocyanins [3,23]. The present findings regarding the degradation of anthocyanins in wines vinified from PEF-treated Merlot grapes as a result of extended storage time have also been observed in wines vinified from PEF-treated Cabernet Sauvignon grapes (5 kV/cm, 3.67 kJ/kg) after 12 months bottle ageing [7] and 14 months ageing in oak barrels [9], both at 18 °C prolonged storage. Moreover, these two previous studies did not observe significant difference in the color intensity between PEF and control wines before or after wine aging [7,9]. It is probably because the color stabilization and co-pigmentation reactions of anthocyanins with other phenolic acids in Cabernet Sauvignon wine is more stable than wines vinified with Merlot varietal.

#### 3.2.3. Reduction of Esters and Acetates Volatiles over Storage Time at Cold Storage

**Figure 4:**
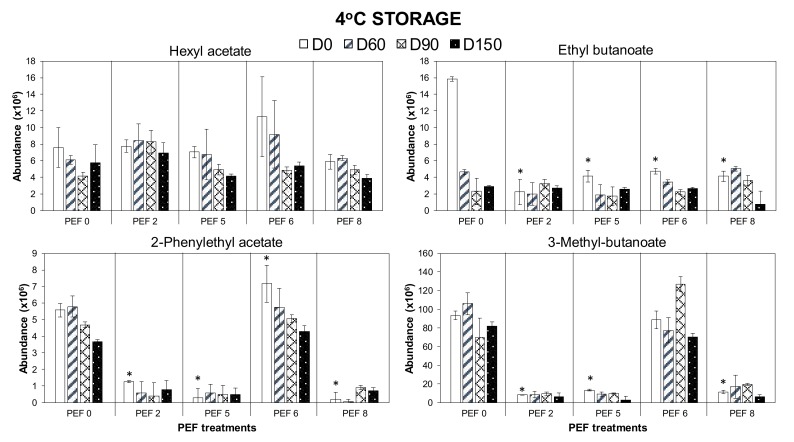
Reduction in the number of selected esters and acetates volatiles in Merlot PEF wine samples over storage time at 4 °C. Data presented as mean ± standard deviation (*n* = 4). Significant difference (*p* < 0.05) comparing the concentration of the analytes between PEF wines (PEF2, 5, 6 and 8) from non-PEF wine (PEF0) at the start of the storage trial (day 0, D0 white bar) are indicated with asterisk (*).

After 150 days of storage at 4 °C, the loss of esters and acetates is among the most dominant change in most wines (Figure 4) except from the PEF2-treated grapes (33.1 kV/cm, 18.90 kJ/kg). In young wines, the presence of these volatile compounds is considered desirable as they contribute towards the fresh and fruity aroma owing to the excess production during yeast fermentation. However, their gradual hydrolysis during aging is unavoidable to eventually reach a balance with respective alcohols and fatty acids, resulting in the loss of freshness in stored or aged wines [25,26,27].

#### 3.2.4. Wine Constituents Identified to be Temperature Sensitive under Extreme Storage Condition of 45 °C 

Storage at elevated temperatures is expected to accelerate various chemical reactions in wines involving color change, loss of phenolic and anthocyanin compounds, reducing volatile compounds that contribute to the freshness of wine aroma, and the formation of new volatile compounds [6,28,29]. A study by Scrimgeour et al. [6] has suggested the ideal storage temperature for red wines intended for consumption is at around 15–20 °C. Further in their study, ambient storage temperature was represented by 25 °C, while both the lowest (4 °C) and highest (45 °C) storage temperatures can be considered extreme. 

**Figure 5:**
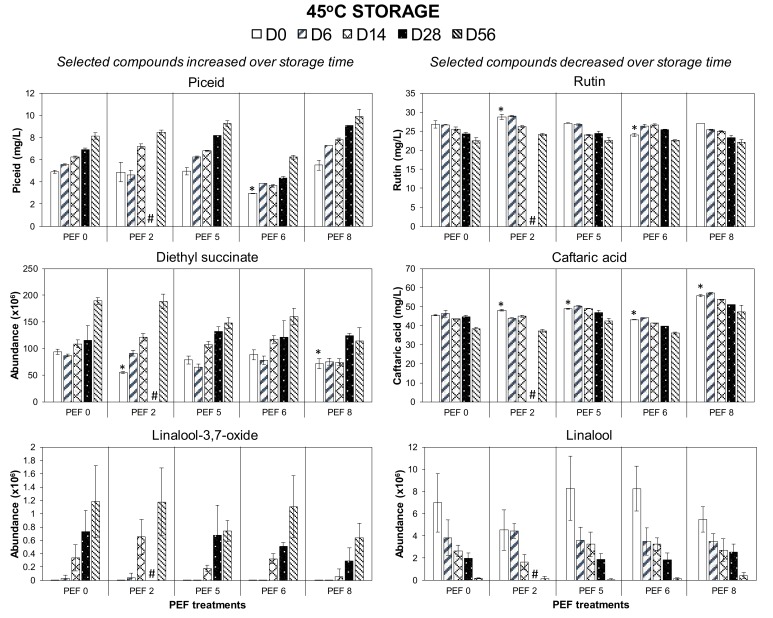
Changes in the amount of selected volatile and phenolic compounds in Merlot PEF wine samples over storage time at 45 °C. Data presented as mean ± standard deviation (*n* = 4). * indicates data not collected. Significant difference (*p* < 0.05) comparing the concentration of the analytes between PEF wines (PEF2, 5, 6, and 8) from non-PEF wines (PEF0) at the start of the storage trial (day 0, D0 white bar) are indicated with asterisk (*). # indicates data not collected.

In the present study, wine storage at 45 °C for up to 56 days affected different classes of volatile and phenolic compounds (Figure 5). With respect to phenolics, piceid was found increase, while caftaric acid and rutin decreased over time. The increase in piceid during storage and its contribution towards the sensorial properties of red wines is poorly understood. It can be speculated that this increase is due to the isomerization of *cis*-piceid to its *trans*- form [30,31]. Moreover, the presence of trace activity of glycosyltransferase enzymes in wine capable of re-glycosylation of resveratrol into piceid during storage may be possible [32,33,34]. 

In the case of caftaric acid reduction, the hydrolysis into its corresponding caffeic acid is expected to be a slow process in conventional ageing and storage of red wines. However, this reaction has been demonstrated to accelerate at high temperatures [4,35,36] and is likely to occur in wines vinified with other PEF-treated red grape varieties when allowed to age up to 14 months at 18 °C [7,9]. A reduction of caftaric acid during storage is expected not to elicit any noticeable difference in the sensory properties of Merlot wine (in terms of bitter and astringent taste properties). This is because the detection threshold of caftaric acid in white wine has been reported to be 150 mg/L [37], which is at least 2–3 times higher than the average concentration reported in this study (Figure 5). 

The loss of rutin (quercetin-3-*O*-rutinoside) might be due to its active co-pigmentation with anthocyanins [38]. Heras-Roger et al. [39] have demonstrated a high correlation of rutin concentration on the co-pigmentation factor in the overall color of red wines. Therefore, changes in rutin content during wine storage plays a larger role in influencing the wine colors, rather than affecting the taste and aroma perception of red wines. Similar to previous studies on red wines vinified with PEF-treated Cabernet Sauvignon [7], a gradual reduction of flavonols, such as quercetin-3-*O*-glucoside, throughout the ageing process in bottle (12 months at 18 °C) appeared to be unavoidable.

With respect to volatile compounds, wines stored in the extreme high temperature in this study were characterized by an increase in the specific ester diethyl butanedioate/diethyl succinate, an increase in the terpene oxide linalool 3, 7-oxide, and a decrease in the latter’s respective terpene alcohol linalool. Diethyl butanedioate/diethyl succinate has been recorded to increase at 40 °C in Aglianco del Vulture red wines [40,41] and is among the esters listed to increase during ageing [42]. An increase in ethyl ester in wine during prolonged storage has been linked to stronger spicy and overripe fruit odors [43]. Linalool 3, 7-oxide is a product of the degradation of linalool, which has been demonstrated to develop at 40 °C in Merlot and Cabernet Sauvignon wines [28,44,45]. Linalool is an important terpene compound that gives a flowery character to Merlot wine variety [13], therefore a loss of this varietal aroma due to prolonged storage of Merlot wine appears to be unavoidable.

#### 3.2.5. Effect of PEF Pre-Treatments on Merlot Grapes on the Evolution of Wine Constituents during Storage

In this study, the finished wines vinified from differently PEF-treated Merlot grapes (PEF0, 2, 5, 6, and 8) varied in their volatile and phenolic compositions, in which a few selected constituents are illustrated in Figure 2, Figure 3, Figure 4 and Figure 5 (refer to D0 data). Therefore, the evolution of some volatile and phenolic markers in the wines distinct to a certain PEF treatment during the prolonged storage at three temperatures can be highly dependent on the initial number of constituents present in the PEF wines at the start of the storage experiment.

For example, prolonged storage at 4 °C for wines vinified using grapes treated with PEF at the highest specific energy (PEF8: 49.40 kJ/kg) has significantly reduced the amount of 11 phenolic compounds classified as anthocyanins, flavonols, and a stilbene (Table 5 and Figure 3). With a higher initial content of anthocyanins throughout the winemaking process, wines from PEF8-treated grapes lost a significant number of anthocyanins during 4 °C storage. As discussed, this reduction could be due to degradation, oxidation, and cleavage self-association, and also co-pigmentation, polymerization, and formation of new pigments [3,22,23,24]. This decrease is aligned with the observation from the study of Puértolas et al. [7] on PEF-treated Cabernet Sauvignon, where a faster reduction rate of monomeric anthocyanins in PEF wines has been reported; although, no significant differences were observed among the wines after storage. In the same study, the rate of reduction for the other non-anthocyanin compounds was found to be either faster or slower in PEF wines compared to the control wines. However, PEF wines typically retained higher amounts of phenolics after storage compared to control wines, which somewhat coincided with our finding on Merlot wines. Hence, a detailed kinetic study of these compounds during storage is needed in future investigations to appropriately compare the thermal stability of the wines tested in this study.

Prolonged storage at 25 °C has generally resulted in a higher content of phenolic acids in PEF stored wines but not in the control wine (Table 1, Table 2, Table 3, Table 4 and Table 5 and Figure 2). This might be due to higher concentration of the precursors present in PEF wines such as the tartaric esters hydrolyzed into free hydroxycinnamic acids, and anthocyanins cleaved into hydroxybenzoic acids during the winemaking process (as discussed in Section 3.2.1). 

As listed in Table 1, Table 2, Table 3, Table 4 and Table 5, more volatile markers distinguished the wines stored at 45 °C: (1) citronellol decreased in stored wines from untreated grapes and those PEF-treated at high specific energies (PEF5 and PEF8, >47 kJ/kg); (2) 2-phenylethyl acetate decreased in stored wines made from untreated grapes and those PEF-treated at low specific energies (PEF2 and PEF6, 16–19 kJ/kg); and (3) more furan compound as a marker in stored wines from untreated and low intensity PEF-treated (PEF2, 33.1 kV/cm and 18.90 kJ/kg) grapes. 

The decrease in citronellol and 2-phenylethyl acetate could be due to increased rate of hydrolysis during storage [25,26]. The differences between the PEF-treatments might have arisen from the differences in the non-volatile components in the wines, especially phenolic composition, which can affect the volatility of these compounds [46]. While both citronellol and 2-phenylethyl acetate have been linked to the flowery and fruity flavors in red wines [47], it is beyond the scope of this study, but of future interest, to understand whether a reduction of these two volatiles contribute significantly to the loss of flowery and fruity flavors in Merlot PEF wines upon storage. 

Moreover, it was found that a higher amount of furfural was found in control wine, while a surge in ethyl 2-furancarboxylate was observed in wine from PEF2-treated grapes as a result of prolonged 45 °C storage. Apart from the occurrence of Maillard reaction and caramelization, the formation and increase in furan compounds in stored wines from untreated and low-intensity-PEF-treated grapes (PEF2) can be due to their increase susceptibility towards oxidation [48,49] or presence of more precursors available in wines from the PEF-treated grapes [26] participating in the aforementioned chemical reactions. Furan related compounds typically contribute to the toasty and caramel-like aroma in wine as a result of significant long periods of storage [50].

## 4. Conclusions

A multiplatform analytical approach combining volatile fingerprinting, phenolic profiling, color and oenological measurements, and strategic analysis of these data through MVDA techniques, namely PCA and PLS-R, were proven to be a robust method to comprehensively visualize and identify important changes in wines vinified with grapes with and without PEF-treatment during their bottle storage at 4 °C, 25 °C, and 45 °C. As a function of storage time, phenolic acids increased, and monomeric anthocyanins decreased along with the dulling of color for all stored wines. These changes were accelerated in higher temperatures for all wines regardless they were vinified with or without PEF-treated grapes. In fact, at 4 °C, the decrease in esters and acetates was shown to be more dominant over the changes in phenolic composition for all stored wines. At high storage temperature of 45 °C, extreme chemical reactions might have occurred leading to increases in the piceid, syringic acid, and linalool 3, 7-oxide concentrations in all wines after storage. Therefore, it can be concluded many of the volatile and phenolic compounds in wines vinified using PEF-treated grapes are evolving or degrading in a similar manner as control wines vinified with untreated grapes, when stored for an extended period of time combined with extreme temperatures. Interestingly, some prominent trends were observed in the wines vinified with PEF-treated grapes at the end of their extended storage time. These include all stored wines produced from grapes PEF-treated with four different processing conditions were shown to favor a high retention of phenolics after 25 °C and 45 °C storage, but stored wines from PEF8-treated grapes induced faster reduction of anthocyanins when compared to wines produced from untreated grapes. It could be because the wines produced from PEF treatment generally have a higher amount of phenolics and anthocyanins; hence their evolution or stability during prolonged storage differed from wines produced from untreated grapes. 2-Phenylethyl acetate and citronellol are the two volatiles where their reductions at the end of 45 °C storage were found to be considerable in wines vinified with grapes PEF-treated at low (16–19 kJ/kg) and high specific (>47 kJ/kg) energies respectively, compared to wines produced using untreated grapes and other PEF-treated grapes. Production of furans was unavoidable for wines at high temperature storage, but the amount of furans were generally lower in wines produced from PEF-treated grapes at higher energy inputs (>47 kJ/kg). The discriminant markers identified in this study can be a reliable representative in differentiating the storage and temperature stability of PEF Merlot wines compared to wines produced from Merlot grapes without subjected to any PEF pre-treatment. While headspace fingerprinting only allows relative compound quantification in this study, it is obvious that future research steps should lead absolute quantification of the selected volatile compounds using pure standards. Furthermore, a follow-up sensory analysis (e.g., descriptive analysis and consumer acceptance testing) should be considered to increase the impact of these candidate markers. 

## Figures and Tables

**Table 1:** Discriminant volatile and phenolic compounds, color, and oenological attributes significantly changed as a function of time at each storage temperature (4 °C, 25 °C, and 45 °C) selected based on variable identification coefficients (VID) coefficient of ≥ |0.800| for wines from untreated Merlot grapes (PEF0) through partial least squares-regression (PLS-R) analysis. The retention index (RI) for volatile compounds is listed. Phenolic compounds were identified and quantified with standards.

4 °C			25 °C			45 °C		
VID	Identity	RI	VID	Identity	RI	VID	Identity	RI
0.894	Gallic acid					0.983	Piceid	
0.891	*trans*-Resveratrol					0.954	Syringic acid	
						0.927	Furfural	1639
						0.897	Diethyl butanedioate	1846
						0.865	Ethyl 2-hydroxy-3-methylbutanoate	1588
						0.859	Linalool 3, 7-oxide	1197
						0.844	pH	
						0.834	*trans*-Coutaric acid	
						0.829	*trans*-Resveratrol	
−0.844	a*		−0.815	Malvidin-3-*O*-(6-*p*-coumaryl)-glucoside		−0.809	(*E*)-Astringin	
−0.849	2-Phenylethyl acetate	1993	−0.826	Peonidin-3-*O*-glucoside		−0.810	Total titratable acidity	
−0.857	Chroma		−0.828	Malvidin-3-*O*-glucoside		−0.822	Linalool	1708
−0.865	1-Heptanol	1610	−0.895	Malvidin-3-*O*-(6-acetyl)-glucoside		−0.859	Caftaric acid	
−0.894	Ethyl butanoate	1095	−0.906	b*		−0.872	Citronellol	1925
−0.906	Acetic acid	1643	−0.933	Isorhamnetin glucoside		−0.900	2-Phenylethyl acetate	1993
−0.916	b*					−0.926	Color intensity	
						−0.934	Astilbin	
						−0.939	Rutin	
						−0.972	Cyanidin-3-*O*-glucoside	
						−0.973	Malvidin-3-*O*-(6-*p*-coumaryl)- glucoside	
						−0.977	Neochlorogenic acid	
						−0.978	Malvidin-3-*O*-(6-acetyl)-glucoside	
						−0.979	Delphinidin-3-*O*-glucoside	
						−0.984	Petunidin-3-*O*-glucoside	
						−0.984	Malvidin-3-*O*-glucoside	
						−0.985	Peonidin-3-*O*-glucoside	

a* (green to red), and b* (blue to yellow).

**Table 2:** Discriminant volatile and phenolic compounds, color, and oenological attributes significantly changed as a function of time at each storage temperature (4, 25, and 45 °C) selected based on VID coefficient of ≥ |0.800| for wines from PEF2-treated Merlot grapes (33.1 kV/cm, 18.90 kJ/kg) through PLS-R analysis. The retention index (RI) for volatile compounds is listed. Phenolic compounds were identified and quantified with standards.

4 °C			25 °C			45 °C		
VID	Identity	RI	VID	Identity	RI	VID	Identity	RI
0.903	Protocatechuic acid		0.974	Gallic acid		0.975	Diethyl butanedioate	1846
			0.829	Ethyl 2-methyl-butanoate	1113	0.953	pH	
						0.944	Nonanal	1555
						0.908	Piceid	
						0.893	Ethyl 2-furancarboxylate	1801
						0.884	3-Hydroxy-2,2,4-trimethylpentyl 2-methylpropanoate	2026
						0.881	2,2,4-Trimethyl-1,3-pentanediol diisobutyrate	2033
						0.867	Linalool 3, 7-oxide	1197
						0.860	Syringic acid	
						0.859	trans-*p*-Coumaric acid	
						0.849	*trans*-Resveratrol	
						0.832	Octanol	1720
						0.826	Butyl ethyl butanedioate	1956
						0.816	Ethyl 3-methylbutyl butanedioate	2051
−0.841	a *		−0.879	Isorhamnetin glucoside		−0.820	2-Phenylethyl acetate	1993
−0.853	Chroma		−0.909	Malvidin-3-*O*-(6-*p*-coumaryl) glucoside		−0.863	Linalool	1708
−0.897	b *		−0.926	Myricetin glucoside		−0.929	Caftaric acid	
−0.911	Kaempferol glucoside		−0.930	Cyanidin-3-*O*-glucoside		−0.938	Color intensity	
			−0.954	Delphinidin-3-*O*-glucoside		−0.938	Malvidin-3-*O*-(6-acetyl)-glucoside	
			−0.958	Malvidin-3-*O*-glucoside		−0.939	Malvidin-3-*O*-(6-*p*-coumaryl) glucoside	
			−0.958	Petunidin-3-*O*-glucoside		−0.951	Delphinidin-3-*O*-glucoside	
			−0.965	Malvidin-3-*O*-(6-acetyl)-glucoside		−0.953	Malvidin-3-*O*-glucoside	
			−0.968	Peonidin-3-*O*-glucoside		−0.953	Rutin	
						−0.954	Petunidin-3-*O*-glucoside	
						−0.954	Peonidin-3-*O*-glucoside	
						−0.967	Cyanidin-3-*O*-glucoside	

a* (green to red), and b* (blue to yellow).

**Table 3:** Discriminant volatile and phenolic compounds, color, and oenological attributes significantly changed as a function of time at each storage temperature (4 °C, 25 °C, and 45 °C) selected based on VID coefficient of ≥ |0.800| for wines from PEF5-treated Merlot grapes (33.1 kV/cm, 47.25 kJ/kg) through PLS-R analysis. The retention index (RI) for volatile compounds is listed. Phenolic compounds were identified and quantified with standards.

4 °C			25 °C			45 °C		
VID	Identity	RI	VID	Identity	RI	VID	Identity	RI
0.908	Ethyl gallate		0.925	Syringic acid		0.977	Piceid	
0.856	Gallic acid		0.896	Caffeic acid		0.936	Syringic acid	
0.848	Caffeic acid		0.884	Gallic acid		0.905	Diethyl butanedioate	1846
0.818	(+)-Catechin		0.873	Ethyl gallate		0.889	1-Octanol	1720
0.810	Astilbin		0.861	Diethyl butanedioate	1846	0.879	pH	
0.808	Caftaric acid					0.867	Dipropyl butanedioate	1105
						0.817	2,6,6-Trimethyl-2-ethenyltetrahydro-2H-pyran	1197
						0.805	Ethyl 3-methyl-butanoate	1136
						0.801	Ethyl 2-hydroxy-3-methylbutanoate	1588
−0.801	Ethyl 3-hydroxy-butanoate	1687	−0.808	Delphinidin-3-*O*-glucoside		−0.810	Citronellol	1925
−0.805	Ethyl 2-hydroxy-4-methyl-pentanoate	1712	−0.811	Malvidin-3-O-(6-*p*-coumaryl) glucoside		−0.827	Linalool	1708
−0.819	3-Methyl-1-pentanol	1462	−0.819	Cyanidin-3-*O*-glucoside		−0.898	Caftaric acid	
−0.821	Octanol	1720	−0.878	Petunidin-3-*O*-glucoside		−0.899	Rutin	
−0.832	Methoxyacetic acid, 3-methylbutyl ester		−0.879	Peonidin-3-*O*-glucoside		−0.966	Malvidin-3-*O*-(6-*p*-coumaryl) glucoside	
−0.832	pH		−0.893	Malvidin-3-*O*-glucoside		−0.981	Malvidin 3-O-(6-acetyl)-glucoside	
−0.833	a*		−0.931	Malvidin-3-*O*-(6-acetyl)-glucoside		−0.987	Petunidin-3-*O*-glucoside	
−0.836	Chroma					−0.988	Malvidin-3-*O*-glucoside	
−0.837	2-Heptanol	1450				−0.988	Delphinidin-3-*O*-glucoside	
−0.846	Protocatechuic acid					−0.990	Peonidin-3-*O*-glucoside	
−0.866	3-Methyl-butanoate	1857				−0.992	Cyanidin-3-*O*-glucoside	
−0.893	(*E*)-Astringin							
−0.906	b*							

a* (green to red), and b* (blue to yellow).

**Table 4:** Discriminant volatile and phenolic compounds, color, and oenological attributes significantly changed as a function of time at each storage temperature (4 °C, 25 °C, and 45 °C) selected based on VID coefficient of ≥ |0.800| for wines from PEF6-treated Merlot grapes (41.5 kV/cm, 16.47 kJ/kg) through PLS-R analysis. The retention index (RI) for volatile compounds is listed. Phenolic compounds were identified and quantified with standards.

4 °C			25 °C			45 °C		
VID	Identity	RI	VID	Identity	RI	VID	Identity	RI
0.961	Gallic acid		0.920	Syringetin glucoside		0.967	Piceid	
0.956	Caffeic acid		0.917	Kaempferol glucoside		0.936	Syringic acid	
0.948	Syringetin glucoside		0.857	*trans*-Coutaric acid		0.932	Dipropyl butanedioate	1105
0.939	(+)-Catechin		0.843	Gallic acid		0.903	2,6,6-Trimethyl-2-ethenyltetrahydro-2H-pyran	1197
0.928	Protocatechuic acid		0.815	Linalool	1708	0.846	1-Octanol	1720
0.915	Myricetin glucoside		0.814	Piceid		0.838	Diethyl butanedioate	1846
0.875	Rutin		0.812	Quercetin		0.825	*trans*-*p*-Coumaric acid	
0.861	*trans*-Resveratrol					0.822	Protocatechuic acid	
0.852	Quercetin					0.819	*trans*-Coutaric acid	
0.832	Neochlorogenic acid							
0.806	Piceid							
−0.830	Isobutyl acetate	1064	−0.801	Isorhamnetin glucoside		−0.828	2-Phenylethyl acetate	1993
−0.833	Acetic acid	1643	−0.814	Neochlorogenic acid		−0.835	Linalool	1708
−0.848	2-Phenylethyl acetate	1993	−0.874	b*		−0.939	Cyanidin-3-*O*-glucoside	
−0.889	Ethyl butanoate	1095	−0.903	Chroma		−0.955	Caftaric acid	
−0.982	a*		−0.904	a*		−0.975	Malvidin-3-*O*-(6-*p*-coumaryl)-glucoside	
−0.988	Chroma		−0.963	Malvidin-3-*O*-(6-*p*-coumaryl)-glucoside		−0.983	Malvidin-3-*O*-(6-acetyl)-glucoside	
			−0.964	Peonidin-3-*O*-glucoside		−0.986	Delphinidin-3-*O*-glucoside	
			−0.977	Malvidin-3-*O*-glucoside		−0.991	Petunidin-3-*O*-glucoside	
			−0.982	Malvidin-3-*O*-(6-acetyl)-glucoside		−0.993	Peonidin-3-*O*-glucoside	
			−0.985	Petunidin-3-*O*-glucoside		−0.993	Malvidin-3-*O*-glucoside	
			−0.987	Delphinidin-3-*O*-glucoside				

a* (green to red), and b* (blue to yellow).

**Table 5:** Discriminant volatile and phenolic compounds, color, and oenological attributes significantly changed as a function of time at each storage temperature (4 °C, 25 °C, and 45 °C) selected based on VID coefficient of ≥ |0.800| for wines from PEF8-treated Merlot grapes (41.5 kV/cm, 49.40 kJ/kg) through PLS-R analysis. The retention index (RI) for volatile compounds is listed. Phenolic compounds were identified and quantified with standards.

4 °C			25 °C			45 °C		
VID	Identity	RI	VID	Identity	RI	VID	Identity	RI
0.946	Gallic acid		0.833	Gallic acid		0.962	Syringic acid	
0.887	Hue angle					0.951	Caffeic acid	
0.884	Caffeic acid					0.925	Piceid	
0.875	*trans*-Coutaric acid					0.872	2,6,6-Trimethyl-2-ethenyltetrahydro-2H-pyran	1197
0.871	Myricetin glucoside					0.855	Dipropyl butanedioate	1105
0.869	L*					0.831	(±)-Eldanolide	1058
0.824	2,2,4-Trimethyl-1,3-pentanediol diisobutyrate	2033						
−0.810	Syringetin glucoside		−0.802	Hue angle		−0.814	Citronellol	1925
−0.811	Hexyl acetate	1404	−0.811	a*		−0.867	Linalool	1708
−0.817	Peonidin-3-*O*-glucoside		−0.816	2-Phenylethyl acetate	1993	−0.872	*trans*-Fertaric acid	
−0.818	Quercetin		−0.821	Chroma		−0.894	Caftaric acid	
−0.818	Malvidin-3-*O*-(6-acetyl)-glucoside		−0.828	Petunidin-3-*O*-glucoside		−0.903	Neochlorogenic acid	
−0.825	Ethyl butanoate	1095	−0.834	Peonidin-3-*O*-glucoside		−0.933	(+)-Catechin	
−0.850	Isobutyl acetate	1064	−0.838	L*		−0.949	Astilbin	
−0.859	(*E*)-Astringin		−0.844	Malvidin-3-*O*-glucoside		−0.952	Rutin	
−0.864	Astilbin		−0.844	Malvidin-3-*O*-(6-*p*-coumaryl)-glucoside		−0.975	Malvidin-3-*O*-(6-*p*-coumaryl)-glucoside	
−0.890	Malvidin-3-*O*-glucoside		−0.881	b*		−0.980	Malvidin-3-*O*-(6-acetyl)-glucoside	
−0.901	Delphinidin-3-*O*-glucoside		−0.889	Malvidin-3-*O*-(6-acetyl)-glucoside		−0.983	Delphinidin-3-*O*-glucoside	
−0.901	Cyanidin-3-*O*-glucoside					−0.983	Petunidin-3-*O*-glucoside	
−0.906	Petunidin-3-*O*-glucoside					−0.985	Malvidin-3-*O*-glucoside	
−0.928	Kaempferol glucoside					−0.986	Peonidin-3-*O*-glucoside	
−0.958	a*					−0.994	Cyanidin-3-*O*-glucoside	
−0.960	b*							
−0.962	Chroma							

L* (lightness), a* (green to red), and b* (blue to yellow).
